# Kisspeptin Suppresses Inflammasome-NLRP3 Activation and Pyroptosis Caused by Hypothyroidism at the Maternal-Fetal Interface of Rats

**DOI:** 10.3390/ijms24076820

**Published:** 2023-04-06

**Authors:** Bianca Reis Santos, Jeane Martinha dos Anjos Cordeiro, Luciano Cardoso Santos, Larissa da Silva Santana, Acácia Eduarda de Jesus Nascimento, Juneo Freitas Silva

**Affiliations:** Centro de Microscopia Eletronica, Departamento de Ciencias Biologicas, Universidade Estadual de Santa Cruz, Campus Soane Nazare de Andrade, Ilheus 45662-900, Brazil

**Keywords:** inflammasome, pyroptosis, NLRP3, thyroid, kiss 1, placenta, decidua, rat

## Abstract

Gestational diseases such as preeclampsia and gestational diabetes cause inflammasome activation and pyroptosis in the placenta and changes in placental kisspeptin levels. Although maternal hypothyroidism also reduces the kisspeptin/Kiss1R system at the maternal-fetal interface, there is still no information on whether this dysfunction causes inflammasome activation and pyroptosis in the placenta or influences the modulatory role of kisspeptin in these processes. This study aimed to evaluate whether hypothyroidism activates the inflammasome-NLRP3 pathway and pyroptosis at the maternal-fetal interface of rats and whether kisspeptin can modulate these processes. Hypothyroidism was induced in Wistar rats by the administration of propylthiouracil. Kisspeptin-10 (Kp10) treatment began on the 8th day of gestation (DG). Gene and/or protein expressions of NLRP3, Caspase 1, IL-1β, IL-18, and Gasdermin D (Gsmd) were evaluated in the deciduae and placentae at the 18th DG. Hypothyroidism increased the decidual and placental stainings of NLRP3, IL-1β, and Gasdermin D, and increased the gene expressions of *Nlrp3*, *Ilβ*, and *Il18* in the placenta and of *Gsmd* in the decidua. Treatment with Kp10 suppressed the increase in NLRP3/*Nlrp3*, IL-1β, *Il18*, and Gasdermin D/*Gsmd* caused by hypothyroidism at the maternal-fetal interface. However, Kp10 increased the placental gene expressions of *Casp1* and *Il1β*. The findings demonstrated that maternal hypothyroidism activated the inflammasome-NLRP3 pathway and pyroptosis at the maternal-fetal interface of rats and that treatment with Kp10 was able to block these processes, thus suggesting that kisspeptin analogues may be promising in the treatment of gestational diseases that involve inflammasome activation and pyroptosis.

## 1. Introduction

Maternal hypothyroidism is one of the most common gestational metabolic disorders and affects around 2–3% of the population [1]. Women with maternal hypothyroidism have an increased risk of miscarriage, premature birth, placental abruption, preeclampsia, gestational diabetes, and intrauterine growth restriction [2,3,4,5,6,7,8], while in hypothyroid female rats the placental development is compromised, with alteration of immunology and trophoblastic endocrine function [7,9,10,11,12,13,14]. In addition, a recent study demonstrated that maternal hypothyroidism also causes oxidative stress and endoplasmic reticulum stress at the maternal-fetal interface of rats [15], suggesting that this cellular stress may result from the failure of intrauterine trophoblastic migration observed in these animals [12].

It has been reported that oxidative stress and endoplasmic reticulum stress can activate not only the classical inflammation pathways [16,17] but also the inflammasome-NLRP3-pyroptosis pathway [18,19,20,21,22,23]. This pathway is also involved in the placental changes seen in preeclampsia, miscarriage, and gestational diabetes [24,25,26,27,28,29], and it involves activation of the intracellular innate immunity receptor NLRP3, with subsequent production of Caspase 1, IL-1β, and IL-18 [19,20,21]. In addition to the exacerbated productions of these inflammatory cytokines, the activation of the inflammasome-NLRP3 pathway can lead to pyroptosis, a form of programmed cell death associated with inflammation mediated by the cleavage of Gasdermin D through Caspase 1 [19,22,30,31]. However, it is unknown whether activation of the inflammasome-NLRP3-pyroptosis pathway is also involved in placental dysfunction caused by maternal hypothyroidism.

We recently demonstrated that maternal hypothyroidism also reduces the expression of kisspeptin and its receptor at the maternal-fetal interface of female rats [32] and that administration of kisspeptin improves fetoplacental development in these animals and suppresses oxidative stress [33]. Pharmacological inhibitors of the inflammasome pathway have broad therapeutic potential for the treatment of diseases that involve activation of this process [21,22,27,29]. Moreover, previous studies have demonstrated the immunomodulatory activity of kisspeptin on monocytes, neutrophils, and regulatory T lymphocytes [34,35,36]. However, there is no information on whether kisspeptin is also capable of modulating the activation of the inflammasome-NLRP3 pathway and pyroptosis.

Thus, the aims of this study were to evaluate whether maternal hypothyroidism activates the inflammasome-NLRP3-pyroptosis pathway in the maternal-fetal interface of rats and to discover the modulatory role of kisspeptin in this pathway.

## 2. Results

### 2.1. Kisspeptin-10 Suppresses Placental Activation of the Inflammasome-NLRP3 Pathway Caused by Maternal Hypothyroidism

**Figure 1:**
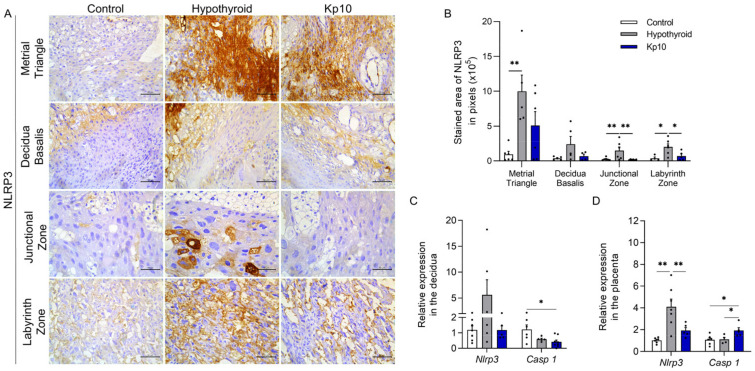
Expressions of NLRP3 and Caspase 1 at the maternal-fetal interfaces of control, hypothyroid, and kisspeptin-10 (Kp10)-treated rats. (**A**) Photomicrographs of the immunohistochemical expression of NLRP3 at the maternal-fetal interface (streptavidin-biotin-peroxidase; Harris hematoxylin; Bar = 50 μm). (**B**) Immunostaining area in pixels of NLRP3 expressions in the metrial triangle, basal decidua, junctional zone, and labyrinth zone (mean ± SEM; *n* = 8). (**C**) Relative gene expression of *Nlrp3* and *Casp 1* in the deciduae (mean ± SEM; *n* = 8). (**D**) Relative gene expressions of *Nlrp3* and *Casp 1* in the placenta (mean ± SEM; *n* = 8). Significant differences were determined by post hoc ANOVA SNK, * *p* < 0.05, ** *p* < 0.01. Kp10 = daily treatment with Kp10; DG = day of gestation.

Activation of the inflammasome-NLRP3 pathway is involved in gestational diseases such as preeclampsia and gestational diabetes. This initially involves activation of the NOD-like pattern recognition receptor, NLRP3, with subsequent expression of Caspase 1 and secretions of IL-1β and IL-18 [21,24,25,27,28,37,38]. As such, this study first set out to evaluate the expressions of NLRP3 and Caspase 1 at the maternal-fetal interfaces of hypothyroid and Kp10-treated rats. Immunostaining of NLRP3 was cytoplasmic and heterogeneous in both the decidual and placental regions. The stained area was greater in the metrial triangle, junctional zone, and labyrinth zone in the hypothyroid group than in the control (Figure 1A,B; *p* < 0.05). Treatment with Kp10 reduced the protein expression of NLRP3 in the junctional zone and labyrinth zone compared to the hypothyroid group (*p* < 0.05), matching the control (Figure 1B, *p* > 0.05). In gene expression, there was no significant difference in *Nlrp43* transcripts in the deciduae between the groups, while the group treated with Kp10 showed a reduction in *Casp1* gene expression compared to the control (Figure 1C; *p* < 0.05). In contrast, similarly to immunostaining, hypothyroidism increased mRNA expression of *Nlrp3* in the placenta compared to the control (*p* < 0.01), while treatment with Kp10 reduced the expression of *Nlrp3* in hypothyroid rats, matching the control (Figure 1D; *p* > 0.05). Contrary to the results for the deciduae, treatment with Kp10 increased the placental gene expression of *Casp1* when compared to the control and hypothyroid groups (Figure 1D; *p* < 0.05).

**Figure 2:**
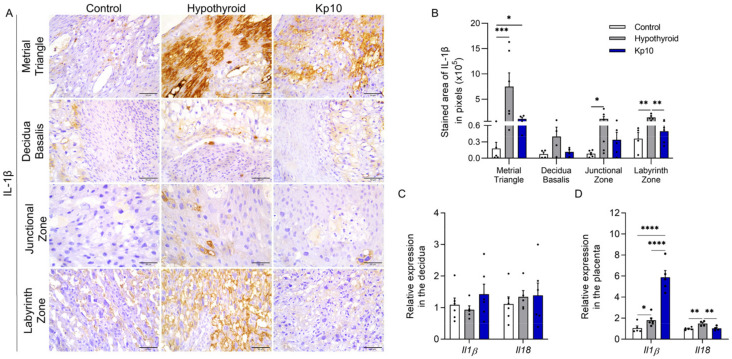
Expressions of IL-1β and IL-18 at the maternal-fetal interfaces of control, hypothyroid, and kisspeptin-10 (Kp10)-treated rats. (**A**) Photomicrographs of the immunohistochemical expression of IL-1β at the maternal-fetal interface (streptavidin-biotin-peroxidase; Harris hematoxylin; Bar = 50 μm). (**B**) Immunostaining areas in pixels of IL-1β expressions in the metrial triangle, basal decidua, junctional zone, and labyrinth zone (mean ± SEM; *n* = 8). (**C**) Relative gene expressions of *Il1β* and *Il18* in the decidua (mean ± SEM; *n* = 8). (**D**) Relative gene expressions of *Il1β* and *Il18* in the placenta (mean ± SEM; *n* = 8). Significant differences were determined by post hoc ANOVA SNK, * *p* < 0.05, ** *p* < 0.01, *** *p* < 0.001, **** *p* < 0.0001. Kp10 = daily treatment with Kp10; DG = day of gestation.

Regarding the expression of IL-1β, immunostaining was also cytoplasmic and heterogeneous throughout the maternal-fetal interfaces of the rats, with greater stained areas in the metrial triangle (*p* < 0.001), junctional zone (*p* < 0.05), and labyrinth zone (*p* < 0.01) in the hypothyroid group compared to the control (Figure 2A,B). Treatment with Kp10, however, reduced IL-1β staining in the labyrinth zones of hypothyroid rats (*p* < 0.01), matching the control (*p* > 0.05). Moreover, no significant difference was observed in the junctional zone compared to the control (*p* > 0.05).

Evaluation of the gene expressions of *Il1β* and *Il18* showed no significant differences between groups in the decidua region (Figure 2C, *p* > 0.05). However, similarly to immunostaining, greater expression of *Il1ß* was observed in the placentae of the hypothyroid group compared to the control (Figure 2D; *p* < 0.05). Furthermore, an increase in expression was observed in the Kp10-treated group compared to the control and hypothyroid groups (*p* < 0.001). Hypothyroidism also increased the placental gene expression of *Il18* compared to the control (Figure 2D; *p* < 0.01), while treatment with Kp10 reduced expression in hypothyroid animals, matching the control (*p* > 0.05).

### 2.2. Kisspeptin-10 Blocks Decidual and Placental Pyroptosis Activation Caused by Maternal Hypothyroidism

**Figure 3:**
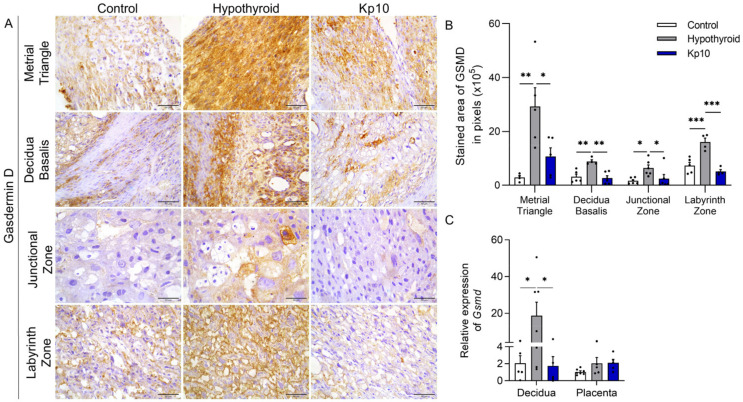
Expression of Gasdermin D at the maternal-fetal interfaces of control, hypothyroid, and kisspeptin-10 (Kp10)-treated rats. (**A**) Photomicrographs of the immunohistochemical expressions of Gasdermin D at the maternal-fetal interfaces (streptavidin-biotin-peroxidase; Harris hematoxylin; Bar = 50 μm). (**B**) Immunostaining areas in pixels of Gasdermin D expression in the metrial triangle, basal decidua, junctional zone, and labyrinth zone (mean ± SEM; *n* = 8). (**C**) Relative gene expression of *Gsmd* in the decidua and placenta (mean ± SEM; *n* = 8). Significant differences were determined by post hoc ANOVA SNK, * *p* < 0.05, ** *p* < 0.01, *** *p* < 0.001. Kp10 = daily treatment with Kp10; DG = day of gestation.

Activation of Caspase 1 in the inflammasome pathway can also result in pyroptosis by cleavage of Gasdermin D [19,39,40], as also observed in the placentae of patients with preeclampsia [30,41,42]. Given the above, this study evaluated the expression of Gasdermin D at the maternal-fetal interfaces of hypothyroid and Kp10-treated rats. The staining of Gasdermin D was cytoplasmic and heterogeneous, with greater expression in the metrial triangle, basal decidua, and the entire placenta (junctional zone and labyrinth zone) of the hypothyroid rats compared to the control Figure 3A,B. Surprisingly, the treatment with Kp10 was able to restore the expression of Gasdermin D in all regions of the deciduae and placentae of hypothyroid rats, reducing its expression and matching the control (*p* > 0.05). Similar to immunostaining, evaluation of transcripts for *Gsmd* showed that hypothyroidism increased its expression in the decidua (Figure 3C; *p* < 0.01), while treatment with Kp10 reduced *Gsmd* expression, matching the control (*p* > 0.05). There was no significant difference in *Gsmd* placental gene expression between the groups (*p* > 0.05).

## 3. Discussion

According to the findings of this study, the gestational dysfunction observed in rats with maternal hypothyroidism involves the activation of the inflammasome-NLRP3-pyroptosis pathway at the maternal-fetal interface. Moreover, treatment with kisspeptin-10 was able to suppress these processes.

Maternal hypothyroidism increased the gene and protein expressions of NLRP3, Caspase 1, IL-1β, and IL-18 in the deciduae and placentae of rats. Moreover, previous studies have demonstrated activation of the inflammasome-NLRP3 pathway in placental alterations observed in preeclampsia, gestational diabetes, and miscarriage [30,42,43]. The activation of this pathway results from the activation of pattern recognition receptors (PRR), mainly toll-like receptors (TLRs), as well as cellular stress [37,38]. Therefore, we suggest that activation of this pathway in the animals of the present study may be associated with the increased placental expression of *Tlr2* demonstrated in hypothyroid rats [12], as well as the oxidative stress, endoplasmic reticulum stress, and immune dysregulation observed in the maternal-fetal interfaces of these animals [12,15,33]. Although the activation of the inflammasome caused by hypothyroidism has been previously described in cardiac tissue, with increased protein expression of NLRP3 and Caspase 1 [44], this is the first study to describe inflammasome complex activation in decidual and placental dysfunction caused by maternal hypothyroidism.

However, not only was inflammasome-NLRP3 activation observed at the maternal-fetal interface of hypothyroid rats, but there was also observed increased decidual and placental expression of Gasdermin D, a key mediator of pyroptosis [45,46]. Overactivation of the inflammasome-NLRP3 complex causes greater activation of Caspase 1, which can lead to cleavage of Gasdermin D, thus resulting in cell death by pyroptosis that triggers the excessive release of inflammatory factors into the extracellular environment [20,21,29,37,46,47]. Furthermore, preeclampsia [30,42] and recurrent miscarriage [43] are associated with the occurrence of pyroptosis in the intrauterine environment. In this regard, this is the first study to demonstrate the occurrence of pyroptosis with the condition of hypothyroidism.

Although changes in plasma and/or placental kisspeptin levels are also observed in maternal hypothyroidism [32], preeclampsia, miscarriage, and gestational diabetes [48,49,50,51,52,53], in the present study, kisspeptin treatment was able to suppress placental and decidual activation of the inflammasome-NLRP3-pyroptosis pathway. Hypothyroid animals treated with Kp10 showed not only reduced expressions of NLRP3/*Nlrp3*, IL-1β, and *Il18* at the maternal-fetal interface, but also of Gasdermin D/*Gsmd*. This demonstrates the modulatory role of kisspeptin on the inflammasome-NLRP3-pyroptosis pathway for the first time. This effect of kisspeptin on this pathway may be the result of its antioxidant and immunomodulatory action [33,34,35,54,55,56,57], as already demonstrated with other inhibitors of the inflammasome-NLRP3 pathway [18,22,58].

However, Kp10 treatment also increased the placental gene expressions of *Il1β* and *Caspase 1*. Although the expressions of IL1β and Caspase 1 are involved in the activation of the inflammasome [18], its increase in the placentae of animals treated with Kp10 is likely to be associated with other metabolic pathways [19,59,60], since treatment with Kp10 blocked the increase in NLRP3 and *Il18*, which are closely associated with inflammasome activation. One hypothesis is that increased placental expression of *Il1β* and *Caspase 1* may result from increased insulin release caused by kisspeptin. Previous studies have already shown that kisspeptin stimulates insulin release [61,62,63,64,65,66] and that mice subjected to a high-fat diet have increased hepatic expressions of *Caspase 1* and *Il1β* [59,67]. However, further studies are needed to prove this hypothesis.

## 4. Materials and Methods

### 4.1. Experimental Design

Adult Wistar rats (200–250 g) were kept in plastic boxes (6 animals/box) with controlled temperature (22 ± 2 °C) and light levels (12:00 h light/12: 00 h dark), with water and feed provided ad libitum. After a 30-day adaptation period, the rats were randomly separated into control (*n* = 13), hypothyroid (*n* = 15), and Kp10-treated hypothyroid (Kp10; *n* = 15) groups. Hypothyroidism was induced 5 days before mating by daily administration via orogastric tube of 4 mg/Kg/day of 6-propyl-2-thiouracil (PTU) (Sigma-Aldrich, St. Louis, MO, USA) diluted in 3 mL of distilled water. The control animals received water as placebo [9,12]

Five days after starting the PTU treatment, five animals from each group were euthanized by decapitation for blood sampling and free T4 dosing to confirm hypothyroidism induction (control, 1.20 ± 0.05 μg/dL; hypothyroidism, 0.47 ± 0.09 μg/dL; Kp10, 0.42 ± 0.08 μg/dL (*p* < 0.01)). Vaginal cytology was performed on the remaining rats and the animals in the proestrus phase were housed with fertile adult male rats for mating. The presence of sperm on the vaginal cytology the following morning confirmed copulation and was defined as day 0 of gestation (0 DG).

Daily intraperitoneal treatment with Kp10 began on the 8th DG with a dose of 8 μg/Kg/day (Cat. No. 4243, Tocris Bioscience, Bristol, UK) [32]. The control and hypothyroid animals received sterile water as placebo. Euthanasia was performed by decapitation on the 18th DG for the collection of deciduae and placentae for immunohistochemistry and qPCR. All experimental procedures were approved by the Ethics Committee for the Use of Animals of the Universidade Estadual de Santa Cruz—UESC (Protocol 002/17).

### 4.2. Necropsy and Collection of Material

At the necropsy, the entire genital tract was collected. Fragments of the placenta and decidua + metrial triangle were dissected and removed from two placental sites/animal and immersed separately in Trizol (Invitrogen, Life Technologies, Carlsbad, CA, USA), followed by freezing in liquid nitrogen and storage at −80 °C for qRT-PCR analysis. The remaining discs (placenta + decidua + metrial triangle) were fixed in paraformaldehyde 4% at 4 °C for 24 h and processed using the paraffin embedding technique for immunohistochemistry. The tissues were dehydrated in a serial solution of 70% to 100% alcohol, with subsequent diaphanization in xylol and impregnation and embedding in paraffin. Tissue sections of 4 μm were obtained by microtomy on silanized polarized slides (StarFrost Polycat, BS, Germany).

### 4.3. Immunohistochemistry

Histological sections of placental discs were subjected to immunohistochemical analysis using antibodies anti-NLRP3 (1:50; sc-134306, Santa Cruz Biotechnology, CA, USA), anti-IL-1β (1:200; sc-12742, Santa Cruz Biotechnology, CA, USA), and anti-Gasdermin D (1:200; sc-393656, Santa Cruz Biotechnology, CA, USA).

The streptavidin-biotin-peroxidase technique was used by the Dako detection system (EnVision™ FLEX+, Mouse, High pH, (Link)). Antigenic recovery was performed by heating in a water bath at 98 °C using EnVision™ FLEX Target Retrieval solution (DM828, Agilent Technologies, Inc., Santa Clara, CA, USA) with pH 6.0. The sections were immersed in hydrogen peroxide solution (3%; H_2_O_2_) with methanol (CH_3_OH) for 30 min to block endogenous peroxidase. The slides were then incubated for an additional 30 min in blocking serum solution (Ultra vision Block, Lab Vision Corp., Fremont, CA, USA) and incubated overnight with the primary antibody. After washing in saline buffered with Tris + Tween 20 (TBS-T; 0.05%; pH 7.6), protein stabilization solution (EnVision™ FLEX+, Mouse (LINKER); ref. SM804) was added, followed by secondary antibody conjugated to streptavidin peroxidase (EnVision™ FLEX/HRP; ref. SM802) for 30 min. The chromogen used was 3′3 diaminobenzidine (EnVision™ FLEX DAB+ Chromogen; ref. DM827), diluted in buffer with H_2_O_2_ (EnVision™ FLEX Substrate Buffer; 1:50; ref. SM803). The sections were counterstained with Harris hematoxylin and the negative control was obtained by replacing the primary antibody with phosphate-buffered solution (PBS) or IgG (Figure S1) [12,68].

A descriptive and quantitative evaluation of the immunohistochemical expression of NLRP3, IL-1β, and Gasdermin D was performed in the layers of the metrial triangle, basal decidua, junctional zone, and labyrinth zone. A quantitative evaluation was performed randomly in six placental discs/group. Images of five random fields in each region of the placental disc were obtained using a Leica DMI 300B photon microscope (Leica Microsystems, Wetzlar, Germany) with 400X magnification. The staining area was determined using WCIF ImageJ^®^ software version 1.41 (Media Cybernetics Manufacturing, Rockville, MD, USA). Color deconvolution and thresholding were determined for each assessed region. Data from each region of the maternal-fetal interface were archived, analyzed, and expressed as staining area in pixels [12,32,33].

### 4.4. qRT-PCR

For the qRT-PCR technique, total RNA was extracted from the decidua (basal decidua + metrial triangle) and placenta using Trizol, according to the manufacturer’s instructions (Invitrogen, Life Technologies, Carlsbad, CA, USA). For the reverse transcription reactions, 1μg of RNA was used with the commercial kit GoTaq^®^ qPCR and RT-qPCR Systems (A6010, PROMEGA, Madison, WI, USA). Target gene transcripts were quantified by qPCR on the Applied Biosystems 7500 Fast Real-Time PCR System (Applied Biosystems, Life Technologies, Carlsbad, CA, USA). For the reactions, 1.5 μL of cDNA, 100 nM of each primer, and 10 μL of the reagent GoTaq^®^ qPCR Master Mix, 2X were used in a final volume of 20 μL of reagent. As a negative control, the DNA amplification mix was used, in which the cDNA sample was replaced by water. Amplifications were performed under the following conditions: enzymatic at 95 °C for 2 min, 40 cycles of denaturation at 95 °C for 15 s, and annealing/extension at 60 °C for 60 s. To evaluate the linearity and efficiency of qPCR amplification, standard curves of all transcripts were generated using serial dilutions of cDNA. The primers for *Nlrp3*, *Il1β*, *Il18*, *Caspase 1*, and *Gasdermin D* were designed based on the *Rattus norvegicus* mRNA sequence (Table 1). Gene expression was calculated by the 2^−ΔΔCT^ method, where the results obtained for each group were compared quantitatively after normalization based on the expression of *Polr2a Rattus norvegicus* [12,33,69].

### 4.5. Statistical Analysis

Significant differences between groups were determined by ANOVA followed by the Student-Newman-Keuls (SNK) test. The data passed the normality (Shapiro-Wilk) and homoscedasticity (Brown-Forsythe) tests of the errors. Data that did not meet the assumptions were subjected to logarithmic transformations (NLRP3, IL-1β, and Gasdermin D immunostaining in the junctional zone and qRT-PCR of *Nlrp3*, *Casp1*, *Gsmd* in the decidua). The data were represented by mean ± standard error of the mean (SEM). For the analyses, the GraphPad Prism 9.0.0 software was used and the differences were considered significant if *p* < 0.05.

## 5. Conclusions

The findings of this study demonstrated that the gestational dysfunction observed in rats with maternal hypothyroidism is associated with activation of the inflammasome-NLRP3-pyroptosis pathway at the maternal-fetal interface, and treatment with Kp10 was able to suppress the activation of this pathway. This is the first study to demonstrate the occurrence of pyroptosis in the condition of hypothyroidism and suggests that kisspeptin analogues may be promising in the treatment of gestational diseases that involve inflammasome activation and pyroptosis.

## Figures and Tables

**Table 1:** List of genes and nucleotide sequence of primers for qRT-PCR.

Gene	Sequence (5′->3′)	Accession Number
*Il18*	F: ACCACTTTGGCAGACTTCACT	NM_019165.1
	R: ACACAGGCGGGTTTCTTTTG	
*Il1β*	F: GCACAGTTCCCCAACTGGTA	NM_031512.2
	R: TGTCCCGACCATTGCTGTTT	
*Nlrp3*	F: CTCTGCATGCCGTATCTGGT	NM_001191642.1
	R: GTCCTGAGCCATGGAAGCAA	
*Casp1*	F: ACAAAGAAGGTGGCGCATTT	NM_012762.2
	R: GTGCTGCAGATAATGAGGGC	
*Gsmd*	F: AAGATCGTGGATCATGCCGT	NM_001130553.1
	F: AAGATCGTGGATCATGCCGT	
*Polr2a*	F: GCTGGACCTACTGGCATGTT	XM_001079162.5
	R: ACCATAGGCTGGAGTTGCAC	

## Data Availability

The data presented in this study are available on request from the corresponding author.

## Supplementary Materials

The following supporting information can be downloaded at: https://www.mdpi.com/article/10.3390/ijms24076820/s1.
