# Supplementary material for: Kisspeptin Suppresses Inflammasome-NLRP3 Activation and Pyroptosis Caused by Hypothyroidism at the Maternal-Fetal Interface of Rats
Source: Int J Mol Sci. 2023 Apr 6;24(7):6820. doi: 10.3390/ijms24076820 (PMC10095583; doi:10.3390/ijms24076820)
Supplement: Supplementary file 1 [file ijms-24-06820-s001.zip › ijms-2085232-supplementary.pdf]

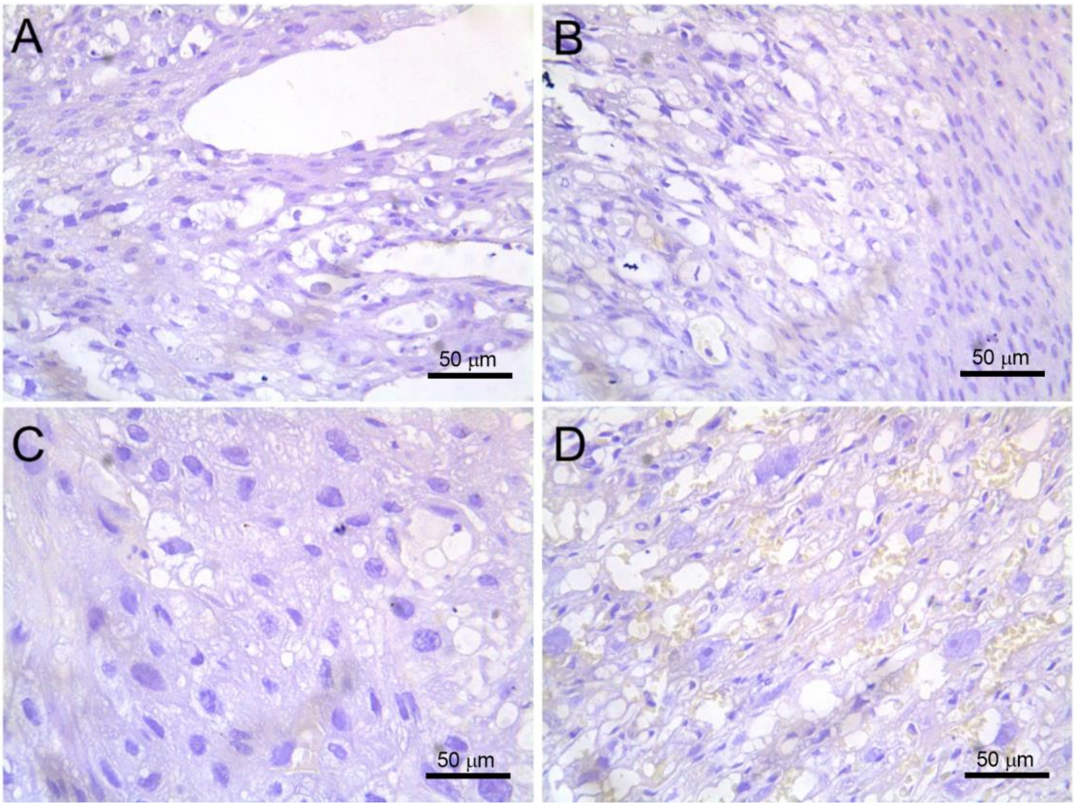

Supplementary Figure S1. Negative control photomicrographs of the immunohistochemistry showing the metrial triangle (A), decídua basalis (B), junctional zone (C), and labyrinth zone (D) (Streptavidin-biotin-peroxidase; Harris Hematoxylin; Bar = 50 µm).
